# Predicting recovery from acid rain using the micro-spatial heterogeneity of soil columns downhill the infiltration zone of beech stemflow: introduction of a hypothesis

**DOI:** 10.1007/s40808-016-0205-8

**Published:** 2016-08-13

**Authors:** Torsten W. Berger, Alexander Muras

**Affiliations:** Department of Forest- and Soil Sciences, Institute of Forest Ecology, University of Natural Resources and Life Sciences Vienna, Peter-Jordan-Strasse 82, 1190 Vienna, Austria

**Keywords:** Beech stemflow, *Fagus sylvatica*, Input–output budget, Soil acidification, Sulfur biogeochemistry

## Abstract

Release of stored sulfur may delay the recovery of soil pH from Acid Rain. It is hypothesized that analyzing the micro-spatial heterogeneity of soil columns downhill of a beech stem enables predictions of soil recovery as a function of historic acid loads and time. We demonstrated in a very simplified approach, how these two different factors may be untangled from each other using synthetic data. Thereafter, we evaluated the stated hypothesis based upon chemical soil data with increasing distance from the stem of beech trees. It is predicted that the top soil will recover from acid deposition, as already recorded in the infiltration zone of stemflow near the base of the stem. However, in the between trees areas and especially in deeper soil horizons recovery may be highly delayed.

## Introduction

Stemflow of beech (*Fagus sylvatica* L.) represents a high input of water and elements, which is why deposition of acidifying substances may be significantly higher close to the stem compared to areas affected by throughfall only (e.g., Chang and Matzner 2000; Kazda and Glatzel 1984; Kazda et al. 1986; Matschonat and Falkengren-Grerup 2000). Enhanced soil acidification around beech stems was also observed by Koch and Matzner (1993) in the Solling area (Germany) and by Sonderegger (1981) and Kazda (1983) in the Vienna Woods. Comparison between chemical parameters of soil from the infiltrations zone of stemflow near the base of the stem and from the between tree area in old beech stands by Lindebner (1990; sample collection in 1984) and Rampazzo and Blum (1992) in the Vienna Woods proved a significant impact of deposition of atmospheric pollutants: soil acidification, heavy metal accumulation, increased total sulfur (S) contents and loss of the base cations calcium and magnesium, especially in the infiltration zone. Since the peak in the 1980s, SO_2_ emissions, the most important precursor of Acid Rain, has declined sharply at an international level (Prechtel et al. 2001). E.g., in Austria, SO_2_ emissions declined from 1980 (385.000 t) to 2013 (17.000 t) by 95 % (Umweltbundesamt 2002, 2015). However, revisiting the Acid Rain topic is worthy, since in many regions mass balance estimates of sulfur are negative due to release of previously-stored sulfur, delaying the recovery of pH of soils and surface waters, depending on soil properties (e.g., see review by Watmough et al. 2005 and references therein; Likens et al. 2002; Likens 2013).

## Hypothesis

We hypothesize that using the micro-spatial heterogeneity of soil columns downhill of a beech stem enables the study of reversibility of soil acidification as a function of historic acid loads (stem area received much higher deposition loads in the past than the between trees area) and time (a “false chronosequence” is expected, since increasing soil solution fluxes due to additional stemflow with decreasing distance from the stem cause a quicker steady state of soil sulfate pools in response to decreasing inputs).

According to Reuss and Johnson (1986), the time required to approach an input–output equilibrium will be highly dependent on soil properties, particularly the sulfate retention capacity, and could be anywhere from a few weeks or months to several decades. From the standpoint of base removal, the time prior to measured increased sulfate soil solution concentrations below the root zone is essentially a “grace period” because cations remain available for plant uptake and recycling in the system. The same authors stated that there is no doubt that sulfate can be immobilized by incorporation in organic matter, but suggested that sulfate adsorption is a more important accumulations mechanism than biological immobilization based upon studies of the early 1980s.

In her article: “Predicting reversibility of acidification: the European sulfur story”, Alewell (2001) concluded that modelling has been seemingly successful in predicting S dynamics in soil solution and stream water by considering inorganic sulfate adsorption and desorption only during the last decades, since deposition was high. However, for soils with low sulfate adsorption capacity and under low sulfate deposition, organic S seems to be a significant source for stream water sulfate and has to be considered in future modelling. The fact that a considerable proportion of atmospherically deposited sulfate is cycled through the organic S pool before being released to soil solution and stream water was indicated by stable S isotopes (^34^S/^32^S ratios; e.g., Alewell et al. 1999; Zhang et al. 1998).

Average annual streamwater outputs of S exceeded S bulk deposition inputs at Hubbard Brook, USA, and this discrepancy may be explained by net desorption of S and net mineralization of organic S largely associated with the forest floor (Likens et al. 2002). Hence, we assume that both processes (desorption/adsorption and mineralization/immobilization) will contribute to the hypothesized quicker steady state of the sulfate input–output balance in (downhill) proximity to the stem basis.

## Results of the hypothesized model using synthetic data

**Fig. 1:**
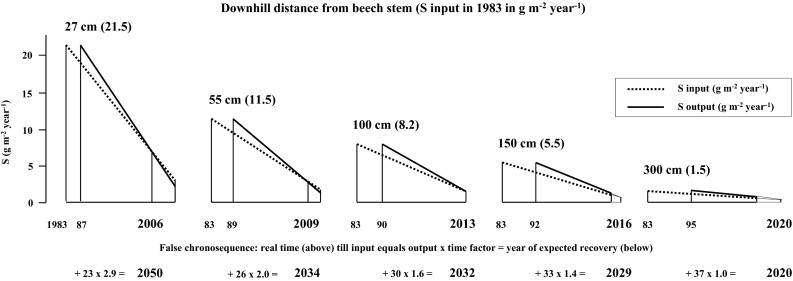
Results of the hypothesized model using synthetic data. The challenge of the model is the differentiation between the two factors (i) historic load and (ii) time. A detailed description is given in the text. E.g., according to these estimates soil recovery (between trees area) is predicted for the year 2034, 2029 and 2020, when S deposition loads amounted 115, 55 and 15 kg S ha^−1^ year^−1^ in 1983, respectively

The challenge of testing the proposed hypothesis is the differentiation between the two factors (i) historic load and (ii) time. We tried to give a simplified demonstration with synthetic data, how these different factors may be untangled from each other in Fig. 1.

**Fig. 2:**
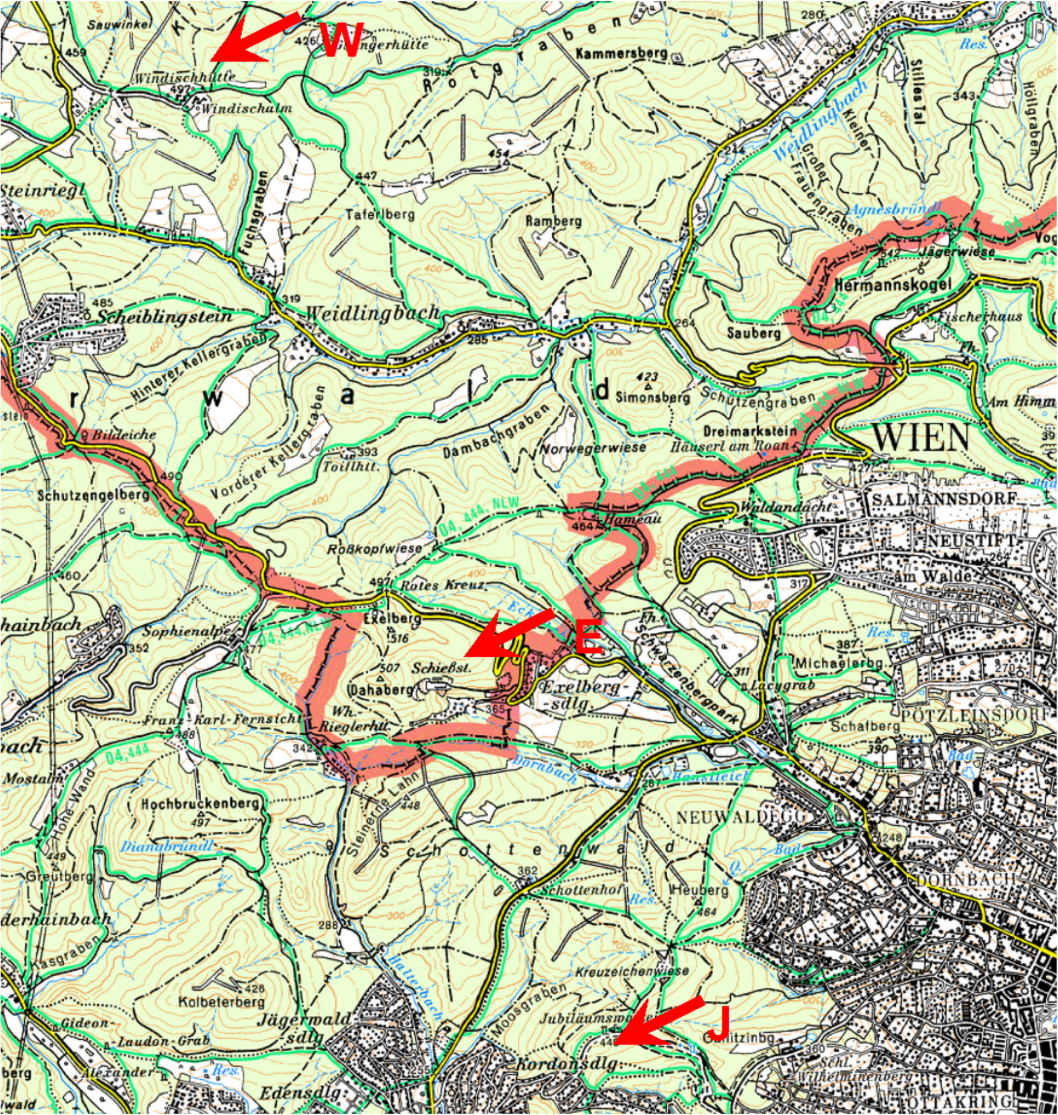
Map of the study sites Jubiläumswarte (J), Exelberg (E) and Windischhütte (W)

Throughfall (TF) and stemflow (SF) of S in 1983 was measured by Sonderegger (1984) at the site Exelberg at the northern border of the City of Vienna (see Fig. 2). Given S flux via SF for the stand area was routed through the soil columns (50, 25, 17, 10 and 0 % for the downhill distances 27, 55, 100, 150 and 300 cm, respectively) assuming a beech crown projection area of 50 m^2^. Input (TF + SF) and output of S (seepage at 50 cm soil depth) in 2013 at 300 cm were taken from Berger et al. (2009) for a comparable beech stand (study site Kreisbach) on Flysch bedrock, approximately 50 km west of the site Exelberg. Output of S in 27 cm (2013) was assumed to equal S input (2013) via SF. The time factor for each downhill distance (false chronosequence) was calculated by given water fluxes (see Berger et al. 2009): (TF + SF)/TF. Maximal S outputs were assumed equal to maximal S inputs but these short term steady state conditions, after relatively constant SO_2_ emissions between 1973 and 1983 (Lefohn et al. 1999), were delayed by 4, 6, 7, 9 and 12 years, corresponding to given time factors. For simplification, declines of S inputs and outputs between 1983 and 2013 were assumed linear and soil recovery was defined: S output ≤ S input. E.g., according to these estimates soil recovery (between trees area) is predicted for the year 2034, 2029 and 2020, when S deposition loads amounted 115, 55 and 15 kg S ha^−1^ year^−1^ in 1983, respectively (Fig. 1).

## Evaluation of the hypothesis

Changes of soil chemistry with increasing distance from the stem of selected beech trees in the Vienna Woods on Flysch were measured by Sonderegger (1981) and Kazda (1983) in the early 1980s and were well documented. At three of these old sites (Jubiläumswarte, Exelberg und Windischhütte; see Fig. 2), soils were resampled and analyzed for pH, total contents of C, N, S and exchangeable cations in 2010 (details about study sites and methods are given by Muras 2012). Flysch consists mainly of old tertiary and mesozoic sandstones and clayey marls. Nutrient release from this bedrock is high and consequently the prevalent humus forms are mull, indicating quick turnover of the forest litter layer (FAO soil classification: stagnic cambisol). Soil cores were sampled down to 50 cm depth at each of the following six distances from one representative beech stem per site: at 55 cm uphill (further labeled −55) and at 27, 55, 100, 150 and 300 cm downhill from the trunk and divided into the mineral soil horizons 0–3, 3–10, 10–20, 20–30 and 30–50 cm depth. At the site Exelberg, top soil (0–3 cm) was sampled in a grid around the beech stem. Mean soil stores (forest floor and mineral soil down to 50 cm depth) at the three sites of C_org_ (80–108), N_tot_ (5–10) und S_tot_ (0.7–1.0; data in t ha^−1^) showed no differences between the individual distances from the stem.

**Fig. 3:**
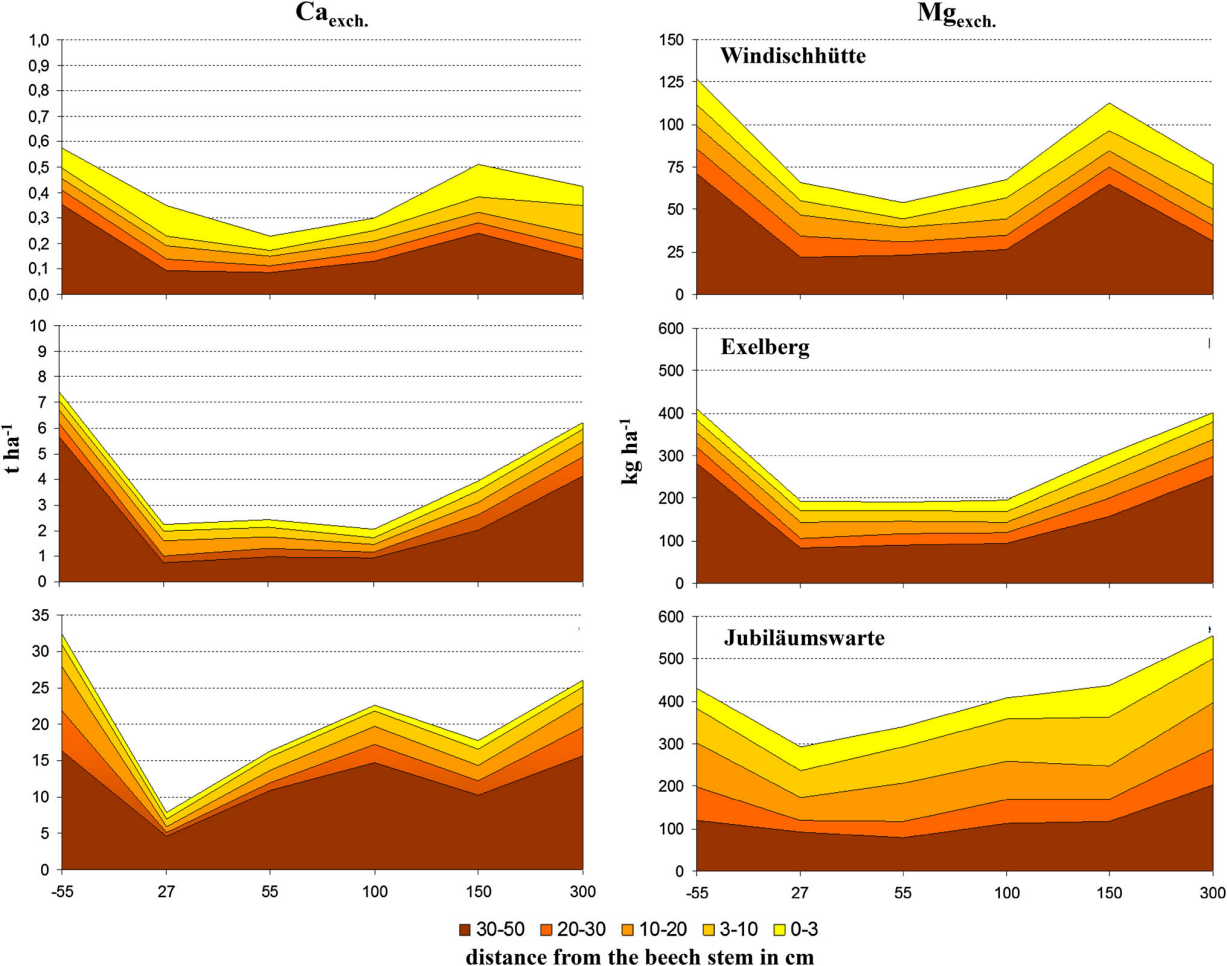
Stores of exchangeable Ca and Mg at the distances −55 (*uphill*) and 27, 55, 100, 150 and 150 cm (*downhill*) of a beech stem for given soil horizons at the study sites Windischhütte, Exelberg and Jubiläumswarte

**Fig. 4:**
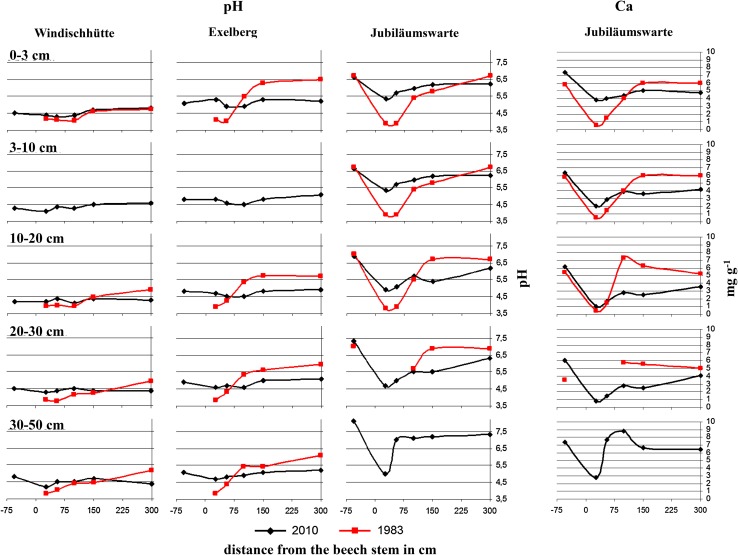
Soil pH (H_2_O) and exchangeable Ca contents (mg g^−1^) at the distances −55 (*uphill*) and 27, 55, 100, 150 and 150 cm (*downhill*) of a beech stem for given soil horizons at the study sites in 1983 and 2010. The so-called 1983 data are modified from Kazda (1983; soil sampling in 1983 at Windischhütte and Exelberg) and Sonderegger (1981; soil sampling in 1980 at Jubiläumswarte)

**Fig. 5:**
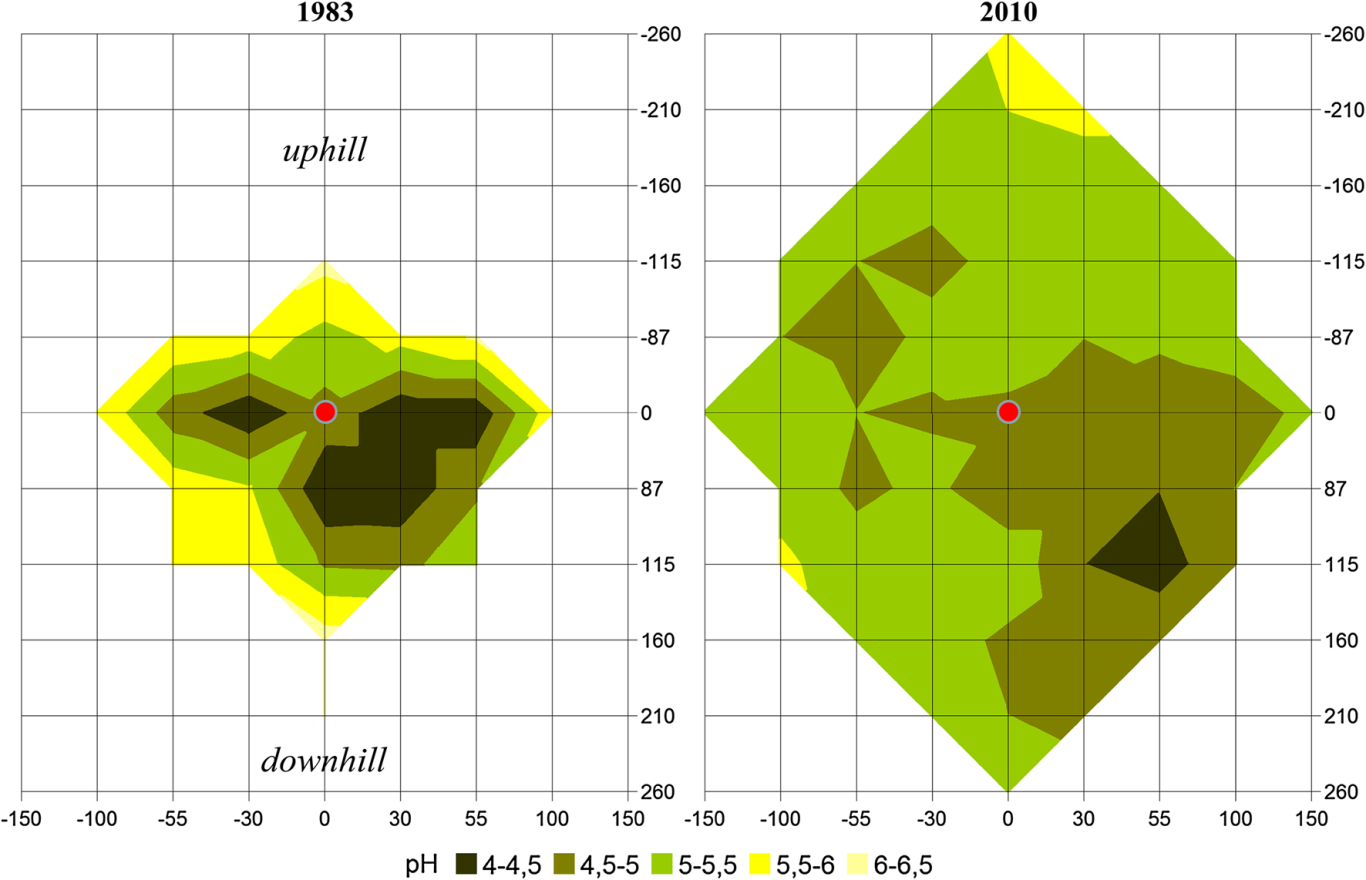
Distribution of soil pH (H_2_O) in top (0–3 cm) soil samples around a beech stem at the study site Exelberg in 1983 (modified from Kazda 1983) and in 2010. The *red dot* marks the stem in the zero-point of a grid. Coordinates are given in cm

The acid input of the last decades was documented by increasing stores of Ca_exch_ and Mg_exch_, respectively, from the base of the stem to the between trees area at 3 m distance (Fig. 3). It is striking that loss of the base cations in the deep soil (30–50 cm), approximate to the stem (downhill), contributed primarily to this overall pattern. While during the 1980s soil pH (H_2_O) near the stem was up to three units lower than distant from the stem, this gradient has flattened down (Fig. 4). Consequently, soil pH close to the stem is currently higher, but at 150–300 cm distances lower than in the 1980s. The same pattern was visible for the top mineral soil (0–3 cm) at Exelberg, sampled in a grid around a beech stand in 1983 (Kazda 1983) and 2010 (Fig. 5). The recent increase of soil pH around the stem basis is related to changing acidity in precipitation, TF and SF. Due to buffering processes within the canopy and deposition of base particulates pH of SF was slightly higher than of adjacent precipitation at the study sites (Muras 2012). While in 1983 SF (pH: 3.82) was significantly more acidic than TF (pH: 4.79) at Exelberg (Sonderegger 1984), these differences were negligible during the summer 2010 at the same site (TF: 6.02; SF: 6.08; Muras 2012). Comparing historic and recent soil pHs indicates that the acidification front-line moved down to deeper soil depths (i.e., recent pHs in the deep soil were lower than in the 1980s; Fig. 4). The best match with these pH trends was found for soil contents of Ca_exch_ (Fig. 4). Hence, we may conclude in accordance to the proposed working hypothesis that soil recovery was speeded up close to the stem, but it does not seem likely that pre-industrial base cation levels will be reached for the whole soil profile within the next future.

According to the hypothesized results with synthetic data, the soil column approximate to the stem basis (27 cm distance, down to 50 cm soil depth) turned from a net S source to a net S sink in 2006 (Fig. 1). This means that in this soil profile neither mineralization nor desorption are currently representing net S sources and both processes must have been accelerated in the past. The following two facts support this conclusion. (i): In the 1980s stores of total S were enriched on Flysch sites in the Vienna Woods as reported by Lindebner (1990; data collection in 1984) and Rampazzo and Blum (1992). Hence, meanwhile, higher amounts of historic S must have been mineralized approximate to the beech stem, since recent stores of total S (0.7–1.0 t ha^−1^) showed no differences between the individual distances from the stem at the three study sites. Higher mineralization rates were probably favoured by higher soil pH, higher base cation- and lower heavy metal contents (Berger et al. 2016). (ii): The adsorbed sulfate fraction (phosphate extractable sulfate-S minus readily available sulfate-S) was significantly reduced next (27 cm) to the stem basis of the study sites according to unpublished data. Accelerated desorption of sulfate in response to input of high stemflow amounts of decreased sulfate concentrations after the end of the 1980s is in accordance to the proposed hypothesis. This mechanism is comparable to “natural water extractions” over historic time-periods.

## Conclusions

**Fig. 6:**
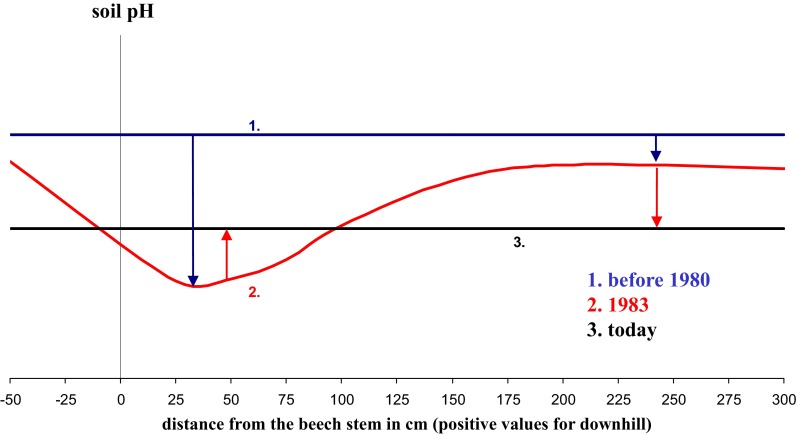
Generalizing spatial soil recovery of the top soil, expressed as pH change

It is concluded that reduced atmospheric sulfate inputs affected soil conditions. It is predicted that the top soil will recover from acid deposition, as already recorded in the infiltration zone of stemflow near the base of the stem. However, in the between trees areas and especially in deeper soil horizons recovery may be highly delayed (Fig. 6).
